# Glycolytic Activities in the Larval Digestive Tract of *Trypoxylus dichotomus* (Coleoptera: Scarabaeidae)

**DOI:** 10.3390/insects5020351

**Published:** 2014-05-05

**Authors:** Noriko Wada, Michio Sunairi, Hirosi Anzai, Ryûtarô Iwata, Akiomi Yamane, Mutsuyasu Nakajima

**Affiliations:** 1 Laboratory of Forest Zoology, Department of Forest Science and Resources, College of Bioresource Sciences, Nihon University, Fujisawa, Kanagawa 252-0880, Japan; E-Mails: wada.noriko@nihon-u.ac.jp (N.W.); iwata@brs.nihon-u.ac.jp (R.I.); akymn22@tbz.t-com.ne.jp (A.Y.); 2 Laboratory of Molecular Microbiology, Department of Applied Biological Science, College of Bioresource Sciences, Nihon University, Fujisawa, Kanagawa 252-0880, Japan; E-Mails: sunairi.michio@nihon-u.ac.jp (M.S.); nakajima@brs.nihon-u.ac.jp (M.N.); 3 Laboratory of Applied Biochemistry, Department of Bioresource Science, Junior College, Nihon University, Fujisawa, Kanagawa 252-0880, Japan

**Keywords:** enzyme, insect, polysaccharide, forest

## Abstract

The larvae of the Japanese horned beetle, *Trypoxylus dichotomus* (Coleoptera: Scarabaeidae: Dynastinae), are an example of a saprophage insect. Generally, Scarabaeid larvae, such as *T. dichotomus*, eat dead plant matter that has been broken down by fungi, such as Basidiomycota. It is thought that *β*-1,3-glucan, a constituent polysaccharide in microbes, is abundant in decayed plant matter. Studies of the degradation mechanism of *β*-1,3-glucan under these circumstances are lacking. In the current study, we sought to clarify the relationship between the capacity to degrade polysaccharides and the food habits of the larvae. The total activities and optimum pH levels of several polysaccharide-degrading enzymes from the larvae were investigated. The foregut, midgut and hindgut of final instar larvae were used. Enzymatic activities were detected against five polysaccharides (soluble starch, *β*-1,4-xylan, *β*-1,3-glucan, pectin and carboxymethyl cellulose) and four glycosides (*p*-nitrophenyl (PNP)-*β*-*N*-acetylglucosaminide, PNP-*β*-mannoside, PNP-*β*-glucoside and PNP-*β*-xyloside). Our results indicate that the digestive tract of the larvae is equipped with a full enzymatic system for degrading *β*-1,3-glucan and *β*-1,4-xylan to monomers. This finding elucidates the role of the polysaccharide-digesting enzymes in the larvae, and it is suggested that the larvae use these enzymes to enact their decomposition ability in the forest environment.

## 1. Introduction

Recently, the investigation of digestive enzymes in the intestinal tract of insects has become increasingly important for the utilization of biomass resources. Reviews of cellulose degrading enzyme in the insects were reported by Martin [1] and Watanabe and Tokuda [2]. In addition, reviews of other polysaccharide digestive enzymes in the internal tract of the insects were reported by Terra and Ferreira, and Ni and Tokuda [3,4]. Within Coleoptera, specific studies of Scarabaeidae have been reported in the following subfamilies: Melolonthinae [5,6,7], Rutelinae [8], Dynastinae [8,9,10,11] and Cetoniinae [8,12,13].

Although most Scarabaeid species (Coleoptera) are saprophagous and/or phytophagous [14,15], their food habits vary considerably among subfamilies and between larvae and adults [16].

The boundary between saprophagy and phytophagy in Scarabaeid larvae is equivocal, since many species of the subfamilies, Scarabaeidae and Aphodiinae, subsist on the dung of grazers, which is thought to be a mixture of degraded plant fiber and microbe mass. Such equivocality is also seen in other subfamilies: species of Rutelinae, Dynastinae and Cetoniinae have been reared on a mixture of animal dung and wood [17,18,19]. One of the most efficient methods for analyzing and characterizing the food habits of Scarabaeidae is to detect larval digestive enzymes. Information on the digestive enzymes of Scarabaeid larvae facilitates the elucidation of their food habits [16] and the establishment of techniques for their industrial utilization [18]. Despite the use of saprophagy in Scarabaeid larvae, research on digestive enzymes in these insects has resulted in the identification of a cellulase and a xylanase that degrade polysaccharides in plant cell walls and an amylase that degrades the starch. Generally, Scarabaeid larvae will eat dead plant matter that has been broken down by fungi, such as Basidiomycota. It is thought that *β*-1,3-glucan, a constituent polysaccharide in microbes, is abundant in such a food source. However, studies of the mechanism of *β*-1,3-glucan degradation under these circumstances are lacking.

The Japanese horned beetle, *Trypoxylus dichotomus* (Linnaeus; formerly *Allomyrina dichotoma*), inhabits the forest floor, where its saprophytic larvae feed on hardwood detritus. It is also known to feed on cultivation logs following the harvest of shiitake mushrooms (*Lentinus edodes*). The larvae are the largest species of the subfamily, Dynastinae, existing on the Japanese mainland. Their large body size and proportional digestive tracts enable the study of their digestive enzymes.

Therefore, the aim of this study was to examine the relationship between the eating habits and digestive enzymes of *T. dichotomus* (L.) larvae to clarify its role as a decomposer in the forest and to investigate the larval use of biomass resources.

## 2. Experimental Section

### 2.1. Insects

Larvae of the Japanese horned Scarabaeid, *T. dichotomus*, were purchased from Tokiwa Shinkô Kôsha (Tokiwa, Tamura City, Fukushima Prefecture, Japan) and reared in the laboratory. We used feeding third-instar larvae as the subjects of this study.

### 2.2. Chemicals

The substrates used to analyze glycanases were as follows. Sodium carboxymethyl cellulose (CMC; Cellogen^®^ BS), soluble starch and chitin (glycol chitin) were purchased from Dai-ichi Kogyo Seiyaku, Wako Pure Chemical Industries and Seikagaku Corp., respectively. Phosphoric acid-swollen cellulose was prepared from absorbent cotton, as described by Green [20]; pachyman (*β*-(1,3)-glucan) was prepared from *Poria cocos*, as described by Cirelli and De Lederkreemer [21], and reduced using NaBH_4_; *β*-(1,4)-xylan (oats spelt xylan; Sigma-Aldrich), which was also reduced by NaBH_4_; *β*-(1,4)-mannan was prepared from the thalli of *Codium latum* (Suringar) using the method described in Iriki and Miwa [22] with slight modifications; and citrus pectin (Wako Pure Chemical Industries) was further purified, as described by Kamimiya *et al.* [23]. The substrates of the glycosidases used in this study, including *p*-nitrophenyl (PNP)-α-glucoside, PNP-*β*-glucoside, PNP-*β*-xyloside, PNP-*β*-mannoside and PNP-*β*-*N*-acetylglucosaminide, were purchased from Sigma-Aldrich. A 10-mM solution of each of these substrates was used as the substrate solution.

After preparing the carbohydrate solutions and suspensions, the concentrations were measured using the phenol-sulfuric acid method [24] and adjusted to 1% (w/v) in terms of the concentration of the main constituent monomer of each sugar, with an exception of 0.04% for chitin. *N*-(2-Acetamido)-2-aminoethanesulfonic acid (ACES) and Coomassie Brilliant Blue-G250 were purchased from Sigma-Aldrich, while tris(hydroxymethyl)aminomethane (Tris), 3,3-dimethylglutaric acid (DGA), 2-amino-2-methyl-1,3-propanediol (AMP), tartaric acid, sodium tartrate and bovine serum albumin were purchased from Wako Pure Chemical Industries.

A 100 mM of ACES buffer (pH 7.5) containing 500 mM NaCl was used for the preparation of gut extracts; a 300-mM GTA buffer consisting of 100 mM DGA, 100 mM Tris, and 100 mM AMP (pH 3.0–11.0), as well as 200 mM tartaric acid/sodium tartrate buffer (pH 1.0–4.0) were used as reaction buffers.

### 2.3. Gut Extracts

**Figure 1:**
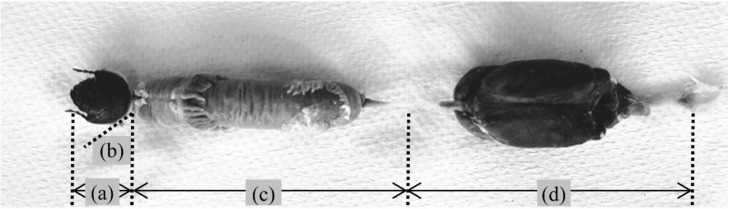
The larval digestive tract of the *Trypoxylus dichotomus* (L.). (**a**) Head; (**b**) foregut; (**c**) midgut; (**d**) hindgut.

Third-instar larvae were wrapped in aluminum foil and placed on ice for 1 h to suppress their activity. The larvae were then dissected, and the guts, including the contents, were removed. The guts were cut into three sections: foregut (including cephalic tissues), midgut and hindgut (Figure 1), in accordance with the definitions of Bayon [11] for *Oryctes nasicornis* (Linnaeus). The sections were stored frozen (below −80 °C) until being further analyzed. Frozen guts with their contents were thawed on ice, cut into pieces using medical scissors, homogenized with a mortar and pestle and suspended in extraction buffer. The suspensions were centrifuged for 15 min at 10,000 × *g*, and the supernatants were used for enzymatic activity assays. Protein concentrations were determined as described by Bradford [25]. The pH was determined by sampling squeezed fluid from each section with a pH meter (F-22; Horiba, Kyoto, Japan). All procedures were carried out at 4 °C, and all solutions were prechilled at 4 °C, unless otherwise stated.

### 2.4. Glycanase Assays

A total of 50 µL of gut extract were mixed with 500 µL of 1% substrate solution, 250 µL of 300 mM GTA buffer and 200 µL of H_2_O and incubated at 30 °C. The concentrations of reducing sugars were determined using the Somogyi-Nelson method [26,27] modified by Anzai *et al.* [28,29]. Briefly, the reaction mixtures were mixed with 1000 µL of Somogyi-Nelson copper reagent, boiled for 15 min, chilled quickly on ice and then mixed with 1000 µL of Nelson reagent. After 15 min, the mixtures were diluted with 3000 µL of H_2_O, and their absorbance at 500 nm was measured using a spectrophotometer (UV1200; Shimadzu, Kyoto, Japan). One unit of glycanase activity was defined as the activity needed to produce reducing sugars equivalent to one micromole (µmole) of monomeric sugar from the substrate polysaccharide per minute.

### 2.5. Glycosidase Assays

A total of 200 µL of reacting fluid containing 5 mM PNP-glycoside, 300 mM GTA buffer and 50 µL of gut extract were incubated at 30 °C. Next, 4 mL of 100 mM sodium carbonate were added to stop the reaction, and then, PNP was quantified based on the absorbance at 420 nm. One unit of glycosidase activity was defined as the activity needed to produce one µmole PNP per minute.

### 2.6. Analysis of the Neutral Sugar Composition of Larval Food

*T. dichotomus* naturally inhabits forests of *Castanopsis sieboldii* or *Fagus crenata*, and its larvae are found in decayed wood or litter, especially wood detritus from *Quercus acutissima* (Japanese chestnut oak) and *Quercus serrata* used for the cultivation of fungi, including shiitake mushrooms [30]. Here, we estimated the neutral sugar composition of *Q. acutissima* as a representative food source of the larvae using previously described methods [31]. Briefly, 0.2-g wood chips were hydrolyzed with 72% sulfuric acid for 1 h at 30 (±0.1) °C. Then, the hydrolysates diluted with water were heated in an autoclave for 1 h at 120 °C and then neutralized to pH 5.5 with a saturated solution of barium hydroxide. The acid hydrolysates were reduced with sodium borohydride; then, reduced monosaccharides, namely alditols, were analyzed by gas-liquid chromatography (GLC) after acetylation. Inositol solution was added as the internal standard. GLC analyses were performed on a Shimazu GC-9A gas chromatograph equipped with an Flame Ionization Detector.

## 3. Results and Discussion

### 3.1. pH of the Gut

The digestive tract of the larvae was divided into three sections: foregut, midgut and hindgut. The pH values of the extracts from the midgut and hindgut were alkaline, with a pH of 10.70 and 8.45, respectively.

Many studies have reported alkaline pH values for the larval midgut of Lamellicornia, including Scarabaeidae [6,7,8,9,11,12,32,33,34,35,36,37,38,39,9,11,12,32]. However, Ricou [40] found that the pH in the midgut and hindgut of the second- to third-instar larvae of *Melolontha melolontha* was almost neutral; only the contents of the midgut and hindgut were alkaline.

### 3.2. The Optimal pH

**Figure 2:**
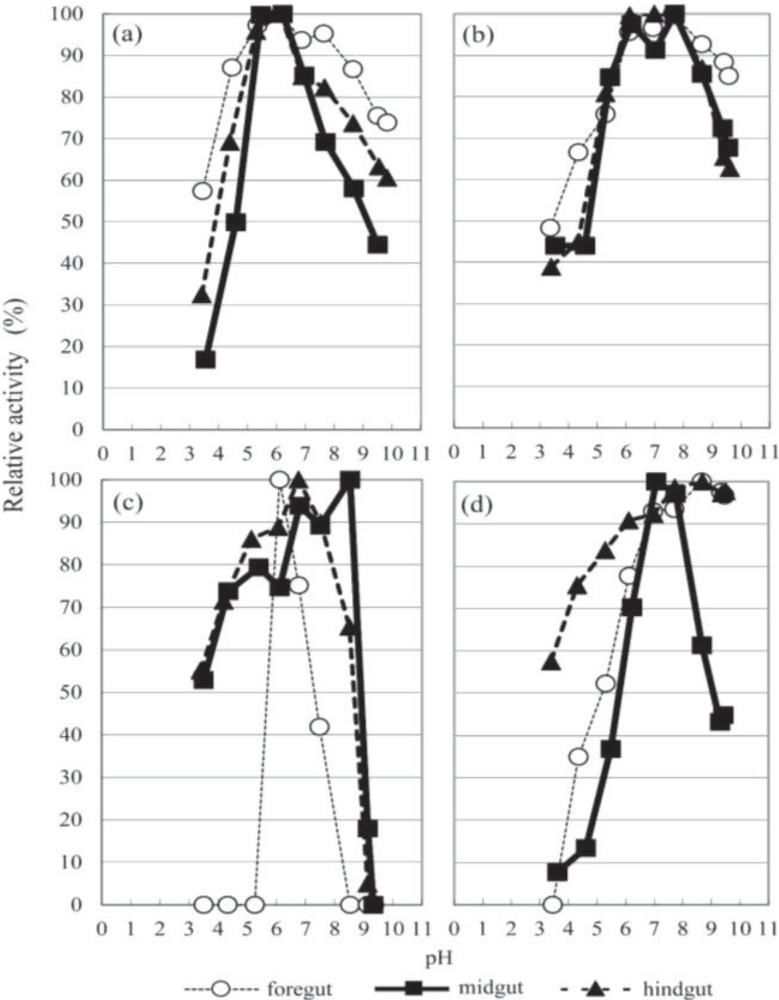
The pH profile of glycanases from the digestive tract of *T. dichotomus* larvae. (**a**) *β*-1,3-glucan; (**b**) *β*-1,4-xylan; (**c**) pectin; (**d**) soluble starch.

**Figure 3:**
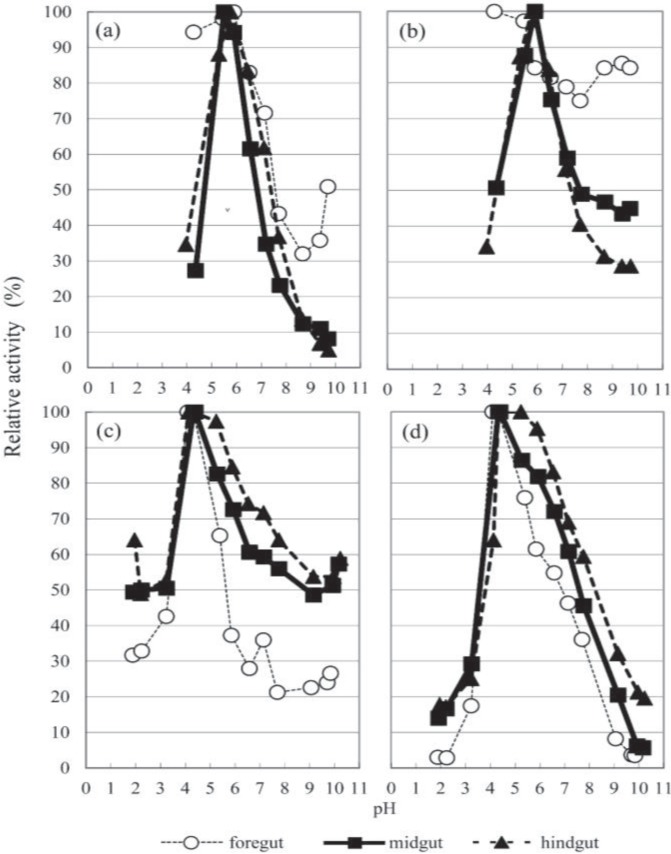
The pH profile of glycosidases from the digestive tract of *T. dichotomus* larvae. (**a**) PNP-*β*-glucoside; (**b**) PNP-*β*-xyloside; (**c**) PNP-*β*-mannoside; (**d**) PNP-*β*-acetylglucosaminide.

The optimal pH values for the enzymes are summarized in Figure 2 and Figure 3 and Table 1 and Table 2. The optimal alkaline pH values observed were in the foregut and hindgut extracts for amylase (pH 8.7) and in the midgut extracts for pectinase (pH 8.5). Conversely, the optimal pH values of the other glycanases were determined to be within a neutral range (6.1–7.6). Amylase activity is optimal at a neutral pH, as has already been presented by Yamane *et al.* [10], and was highest in the midgut, where the pH was, in general, strongly alkaline (Figure 2). Schlottke [12] reported that in *Cetonia aurea* larvae, the optimal pH range of amylase was 6.5–8.5, which was less alkaline than the actual pH value of the midgut (11.0–11.5), and he ascribed this difference in pH to the neutralization effect of the food ingested. The activity of *β*-xylosidase in the foregut extract was too weak to determine its pH dependency. The optimum pHs of *β*-glucosidase and *β*-xylosidase were within the range 5.5–5.9, whereas those of *β*-mannosidase and *β*-*N*-acetyl-glucosaminidase were within the range 4.1–5.2 (Figure 3).

The optimum pH values for glycanase and the corresponding glycosidases, namely *β*-(1,3)-glucanase and *β*-glucosidase, *β*-(1,4)-xylanase and *β*-xylosidase, were acidic. However, the glycanases retained higher activities at an alkaline pH than their corresponding glycosidase. We detected two peak optimal pH values for *β*-(1,4)-xylanase in the midgut extract, at 6.2 and 7.6. It is likely that the acidic peak of xylan was affected by *β*-xylosidase. A previous study reported a double peak in the hemicellulose (including xylose and mannose) of Japanese red pine in *Shirahoshizo rufescens* Roelofs larvae [41].

### 3.3. Glycanase Activities

We examined the glycanases using eight different substrates: CMC, phosphoric acid-swollen cellulose, soluble starch, chitin, pachyman (*β*-(1,3)-glucan), *β*-(1,4)-xylan, *β*-(1,4)-mannan and pectin. Figure 2 shows the pH dependence of these strong glycanases in the extracts from the foregut, midgut and hindgut; their total and specific activities at an optimum pH are summarized in Table 1.

**Table 1:** Glycanase activities from the larval digestive tract of the *T. dichotomus.*

Organ	Substrate	Optimum pH	Total Activity (mU)	Specific Activity	
				(mU/mg Protein)	(mU/g Organ)
Foregut	CM-cellulose	ND ^a^	188 ^b^ ± 25 ^c^	4.79 ± 0.55	164 ± 23
	*β*-1,3-glucan	6.15	194 ± 24	4.95 ± 0.69	169 ± 26
	*β*-1,4-xylan	7.63	1733 ± 415	44.25 ± 11.37	1514 ± 9
	Pectin	6.11	2540 ± 360	64.86 ± 8.20	2219 ± 8
	Soluble starch	8.66	127 ± 26	3.23 ± 0.63	111 ± 19
Midgut	CM-cellulose	ND ^a^	319 ± 28	8.11 ± 0.77	52 ± 6
	*β*-1,3-glucan	6.22	3066 ± 220	77.95 ± 4.15	502 ± 36
	*β*-1,4-xylan	6.18, 7.69	7094 ± 543	180.36 ± 16.14	1162 ± 3
	Pectin	8.53	2982 ± 261	75.80 ± 7.18	488 ± 1
	Soluble starch	7.07	13,520 ± 569	343.74 ± 13.19	2214 ± 85
Hindgut	CM-cellulose	ND ^a^	80 ± 14	5.85 ± 1.02	16 ± 4
	*β*-1,3-glucan	6.14	856 ± 87	62.63 ± 6.94	170 ± 20
	*β*-1,4-xylan	6.98	2039 ± 311	149.19 ± 27.82	404 ± 8
	Pectin	6.76	2836 ± 105	207.51 ± 13.08	562 ± 4
	Soluble starch	8.69	145 ± 36	10.61 ± 3.02	29 ± 8

^a^ Not detected; ^b^ total activity for the carboxymethyl cellulose (CMC) degradation was measured using GTA buffer at pH 7.0; ^c^ SE, standard error (*n* = 6); the organ weight used is as follows: foregut, 0.7759–1.8733 g; midgut, 3.7972–8.7650 g; hindgut, 3.1010–7.0759 g.

**Table 2:** Glycosidase activities from the larval digestive tract of the *T. dichotomus.*

Organ	Substrate	Optimum pH	Total Activity (mU)	Specific Activity	
				(mU/mg Protein)	(mU/g Organ)
Foregut	PNP-*β*-glucoside	5.89	5.09 ^b^ ± 0.83 ^c^	0.13 ± 0.02	4.4 ± 0.83
	PNP-*β*-xyloside	N.D. ^a^	3.10 ± 0.38	0.08 ± 0.01	2.7 ± 0.41
	PNP-*β*-mannoside	4.10	34.40 ± 4.25	1.75 ± 0.61	24.4 ± 3.61
	PNP-*β*-*N*-acetylglucosaminide	4.10	1397.83 ± 127.30	71.08 ± 7.56	992.5 ± 99.64
Midgut	PNP-*β*-glucoside	5.50	94.70 ± 3.21	2.41 ± 0.13	15.5 ± 0.81
	PNP-*β*-xyloside	5.89	25.91 ± 4.23	0.66 ± 0.09	4.2 ± 0.63
	PNP-*β*-mannoside	4.33	301.72 ± 50.75	18.03 ± 8.35	43.1 ± 8.91
	PNP-*β*-*N*-acetylglucosaminide	4.33	702.00 ± 51.74	41.95 ± 4.12	100.3 ± 14.39
Hindgut	PNP-*β*-glucoside	5.77	59.04 ± 3.80	4.32 ± 0.38	11.7 ± 0.54
	PNP-*β*-xyloside	5.77	17.20 ± 2.59	1.26 ± 0.18	3.4 ± 0.48
	PNP-*β*-mannoside	4.13	91.18 ± 11.74	8.29 ± 1.18	15.2 ± 3.60
	PNP-*β*-*N*-acetylglucosaminide	5.24	198.57 ± 45.04	18.05 ± 4.09	33.2 ± 13.76

^a^ Not detected; ^b^ total activity for xyloside was measured using GTA buffer at pH 6.0; ^c^ SE, standard error (*n* = 6); the organ weight used is as follows: foregut, 0.7759–1.8733 g; midgut, 3.7972–8.7650 g; hindgut, 3.1010–7.0759 g.

Overall, glycanase activity was higher in the midgut than in the fore- or hind-gut. Among the polysaccharides used as substrates, the activity was highest using soluble starch, followed by *β*-(1,4)-xylan, *β-*(1,3)-glucan and pectin. In contrast, weak activity was detected against CMC, and little or no hydrolyzing activity was observed using phosphoric acid-swollen cellulose, *β*-(1,4)-mannan and chitin.

The cellulase activity of CMC was so weak that its pH dependence or optimum pH could not be determined. Total activity for CMC degradation was measured using GTA buffer at pH 7.0. In terms of polysaccharide-degrading activity, the amylase activity of the midgut was remarkably high: about 90 times the activity of the hindgut. Moreover, the *β*-1,3-glucanase and *β*-1,4-xylanase activities of the midgut were approximately three to four times those of the hindgut. However, the total activity of pectinase did not change with the gut section analyzed. Amylase showed the strongest activity among the glycanases in extracts from all three gut sections, followed by *β*-(1,3)-glucanase, *β*-(1,4)-xylanase and pectinase. The very high activity of amylase in *T. dichotomus* is comparable with data for *Melolontha vulgaris* (Melolonthinae) from Courtois *et al.* [42] and for *Cetonia* and *Potosia* (Cetoniinae) from Schlottke [12]. However, it conflicts with the results for *Xylotrupes gideon* (Dynastinae) given by Mishra and Sen-Sarma [8], who reported the absence of amylase in the *Xylotrupes* hindgut.

It is also inconsistent with the results for *Popillia japonica* (Rutelinae) by Swingle [32] and different from the results found for *O. nasicornis* (Dynastinae) by Wiedemann [43]. Only trace cellulase activity for the degradation of CMC was detected.

These results are in agreement with those of Debris *et al.* (*Polyphylla fullo*: Melolonthinae) [5], Courtois and Chararas (*Melolontha vulgaris*) [44], Soo Hoo and Dudzinski (*Sericesthis geminata*: Melolonthinae) [6] and Cazemier *et al.* (*Pachnoda marginata*: Cetoniinae) [13], but are inconsistent with those of Bayon [11], who reported an absence of cellulase for CMC degradation in *O. nasicornis*.

The possibility that an enzyme, such as *β*-1,3-glucanase, acts on CMC is worth considering. There is certainly a precedent for this; for example, exo-cellulase could also act on barley glucans. Under normal conditions, barley glucans are hydrolyzed by endo-type, but not exo-type, cellulases. However, the structural characteristics of barley glucans allow hydrolysis by exo-cellulases.

According to reports on the digestion of Scarabaeid larvae, xylanase activity in Scarabaeidae varies considerably by species: *Aphodius rufipes* (animal dung feeder; Aphodiinae) was reported by Holter [45] not to possess xylanase; in the third-instar larvae of *Costelytra zealandica* (soil grub: Melolonthinae), Bauchop and Clarke [46] detected xylanase activity in only the hindgut, which they assumed to be of bacterial origin.

Among several Scarabaeids, Mishra and Sen-Sarma [8] detected xylanase activity only from *Anomala polita* (soil grub; Rutelinae) larval gut contents and not from either cetonine or dynastine species. Cazemier *et al.* [13] detected xylanase activity in both the midgut and hindgut of *P. marginata*, a detritus feeder.

Although the optimal pH of the glycanases in the extracts was not identical to the pH of the gut, the activities of these glycanases at the gut pH were greater than 50% of their maximum levels, suggesting that these glycanases are quite active in the digestive tract of *T. dichotomus*.

### 3.4. Glycosidase Activities

Among the five kinds of PNP-glycosides tested, the highest glycosidase activities were detected against PNP-*β*-*N*-acetylglucosaminide, followed by PNP-*β*-mannoside, PNP-*β*-glucoside and PNP-*β*-xyloside, and no activity was detected against PNP-α-glucoside. The total/specific activities at the optimum pH are shown in Table 2.

The total activity for xyloside was measured using GTA buffer at pH 6.0. In *β*-*N*-acetylglucosaminidase, the total activity in the foregut was remarkably high at approximately twice the activity in the midgut and seven times that in the hindgut. Moreover, the total activities of *β*-glucosidase and *β*-xylosidase in the midgut were about 1.5 times that in the hindgut, and *β*-mannosidase activity in the midgut was approximately 3.3 times that in the hindgut. With the exception of *β*-*N*-acetylglucosaminidase, the specific activities of glycosidases in the midgut were higher than those in the hindgut.

The highest activities of *β*-glucosidase, *β*-mannosidase and *β*-xylosidase were detected in midgut extracts, but the highest activity of *β*-*N*-acetylglucosaminidase was identified in the foregut extract.

We deem this to be commonly applicable to all insects or at least to Scarabaeidae, including *T. dichotomus*. We detected *β*-glycosidase, *β*-*N*-acetylglucosaminidase, *β*-glucosidase, *β*-mannosidase and *β*-xylosidase activity in the larvae, with optimum pH values that were slightly acidic, despite the alkaline environment. Their pH profiles have shapes that are typical for enzyme activity, and approximately 50% of the maximum activities of *β*-mannosidase and *β*-xylosidase and less than 20% of the maximum activities of *β*-*N*-acetylglucosaminidase and *β*-glucosidase took place *in situ* (at pH 10). The inconsistency between the actual pH of the environment where an enzyme is found and its optimal pH range does not seem unusual and may be a physiological compromise.

In addition, a previous study [38] suggested that the inconsistency between the optimal and actual pH might be due to the evolution of *T. dichotomus* larvae.

### 3.5. Neutral Sugar Composition of Larvae Food

Since *T. dichotomus* larvae can feed on chips of *Q. acutissima*, the neutral sugar composition of the chips was determined. Glucose was the dominant component, amounting for as much as 65.4% of the total neutral sugar content. Xylose was the dominant component of hemicellulose (12.9%), followed by arabinose (3.1%) and galactose (4.2%).

In this study, we measured the enzymatic degradation of *β*-1,3-glucan, *β*-1,4-xylan and pectin. Xylan is a major hemicellulose of hardwood and not major in softwood. The major hemicellulose of softwood is glucomannan. As expected, the digestive tract of the larvae possesses strong *β*-(1,4)-xylanase activity, and *β*-(1,4)-mannanase was not detected. These results indicated that the composition of “hemicellulases” in the digestive tracts were dependent on the food habit of the insects. The habitat of *T. dichotomus* larvae is the forest floor, where it feeds on hardwood detritus; the larvae have also been observed feeding on cultivation logs following the harvest of shiitake mushrooms. *β*-1,3-glucan is a component of Basidiomycetes, such as shiitake mushrooms, but it is not a natural component of wood. Cultivation logs that have been used to grow shiitake mushrooms contain *β*-1,3-glucan. As a result of its feeding activity, strong *β*-1,3-glucan degrading activity was detected in the larvae, and the larvae likely ingest *β*-1,3-glucan along with other polysaccharides while feeding. The results presented herein suggest a relationship between *β*-1,3-glucan degrading activity and larval feeding habits.

However, the origin of the enzymes is unclear. It is possible that these activities were already present in feeding substrates (decayed wood or litter) that contained abundant microorganisms and basidiomycetes. The foregut contains higher *β*-*N*-acetylglucosaminidase activity than the midgut or hindgut. However, the foregut is a small, short organ, and so, feeding substances pass through it rapidly. Larvae have also been observed around shiitake mushroom cultivation logs. Basidiomycetes, such as shiitake mushrooms, contain cellulase and *β*-1,3-glucanase [47]. Therefore, if the feeding substances include basidiomycetes, cellulase activity is likely to be detected in the intestines of larvae. However, in the present study, only very low cellulase activities were detected in all parts of the larval gut. In addition, we previously isolated xylanolytic bacteria [48] from alkaline medium. This suggests that the optimal pHs of these enzymes are alkaline. Soils, such as humus, are usually acidic, and so, the enzyme is unlikely to be active under those conditions. Taken together, these data suggest that the larvae might secrete the digestive enzymes.

Furthermore, it is necessary to determine the conditions of larval intestines and intestinal flora to clarify the function of larvae in forest ecosystems. A recent study reported that insects, such as those in the order, Coleoptera, possess glycanase genes [49,50,51]. This suggests that it is possible that *T. dichotomus* also contains such glycanase genes.

## 4. Conclusions

In this study, we characterized the glycanases and glycosidases in the gut of *T. dichotomus* larvae, showing their distribution in the gut and the pH profiles of their activities, thereby affording some insight into the relationship between the polysaccharide-degrading capacity and feeding habits of this insect.

Although amylase had the highest activity among the glycanases from the gut of *T. dichotomus*, its corresponding glycosidase, α-glucosidase, was not detected. This suggests unusual metabolic pathways for starch in the larvae. In contrast, higher *β*-mannosidase and *β*-*N*-acetylglucosaminidase activities were detected, although their corresponding glycanases, mannanase and chitinase, respectively, were not detected, which suggests that these enzymes have functions other than polysaccharide metabolism.

The digestive tract of *T. dichotomus* larvae is equipped with a full enzymatic system for degrading *β*-1,3-glucan and *β*-1,4-xylan to their respective monomers. This finding highlights the role of polysaccharide digestive enzymes in the larvae, which are decomposers in forest ecosystems.
